# The environmental impact of pediatric celiac disease diagnosis and follow‐up

**DOI:** 10.1002/jpr3.70048

**Published:** 2025-06-17

**Authors:** Mario Brusco, Sara Trivellini, Rita Cozzali, Andrea Brusaferro, Olivia Morelli, Rachele Simonte, Giuseppe Di Cara, Francesco Valitutti

**Affiliations:** ^1^ Department of Surgical and Biomedical Sciences, Paediatric Clinic, “S. Maria della Misericordia” University Hospital University of Perugia Perugia Italy; ^2^ Paediatric and Neonatology Unit Foligno Hospital Foligno Italy; ^3^ Digestive Endoscopy Unit “Santa Maria della Misericordia” University Hospital Perugia Italy; ^4^ Department of Medicine and Surgery, Anesthesia, Analgesia and Intensive Care, “S. Maria della Misericordia” University Hospital University of Perugia Perugia Italy

**Keywords:** adolescents, biopsy, celiac disease, children, carbon dioxide, endoscopy

## Abstract

**Objectives:**

To evaluate carbon dioxide (CO_2_) footprint of celiac disease (CeD) diagnostic guidelines and follow‐up practices for children/adolescents.

**Methods:**

Two‐hundred and thirty‐six patients diagnosed and followed up for CeD in Umbria region during 2020–2023 were included in this retrospective study. Patients were divided in two groups: Group 1 included patients diagnosed by duodenal biopsies (total: 43), while Group 2 included no‐biopsy patients (total: 193). Transport emissions of CO_2_ per kilometer traveled by a diesel car was estimated as 171 g/km. CO_2_ cost was estimated as 22 kg for each anesthesia and as 5.4 kg for each upper GI endoscopy.

**Results:**

The median CO_2_ cost/patient/year in Group 1 was 397.9 kg, while the median CO_2_ cost/patient/year in Group 2 was 57.2 kg (*p* < 0.001). As regards the follow‐up of these children, we estimated a median CO_2_ amount of 39.3 kg produced per year by car emission and there was no difference between the two groups (Group 1 40.5 kg vs. Group 2 38.1 kg; *p*:ns).

**Conclusions:**

The no‐biopsy approach for the CeD diagnosis strongly decreases the CO_2_ emissions. Whether implementing telemedicine, handing over to primary care or reducing outpatient consultations for follow‐up will be feasible and environmentally more sustainable should be evaluated.

## Abstract (highlights)

**What is Known:**

- Healthcare systems are among the least green sectors and represent a constant threat to humans as regards greenhouse gas emissions, accounting approximately for 4.6% of global greenhouse gas (GHG) emissions.
- Endoscopy is the third largest generator of waste after anesthesiology and intensive care.

**What is New:**

- The non‐biopsy approach for the diagnosis of CeD reduces health system costs and has a conspicuous environmental benefit by decreasing CO_2_ emissions.
- Whether implementing telemedicine for follow‐up will be feasible and environmentally more sustainable should be evaluated.

## 1 INTRODUCTION

One of the challenges of our century is the global climate change. The atmosphere is warming due to increased greenhouse gas (GHG) emissions,^1^, ^2^ especially carbon dioxide (CO_2_). Human activities produce CO_2_, and this is the main culprit for thermal energy retention in the atmosphere.^3^, ^4^ It has been increasingly acknowledged that worldwide healthcare systems are among the least green sectors and represent a constant threat to humans as regards greenhouse gas emissions, accounting approximately for 4.6% of global GHG emissions.^5^ Endoscopy is the third largest generator of waste after anesthesiology and intensive care.^6^, ^7^

For these reasons, the European Society of Gastrointestinal Endoscopy (ESGE) released in 2022 a Position Statement where it is advocated the need of an urgent shift to reduce the carbon footprint of gastrointestinal (GI) endoscopies.^8^

Considering that pediatric endoscopy should be necessarily performed under anesthesia,^9^, ^10^ the cumulative CO_2_ cost of this sector should not be neglected.

Celiac disease (CeD) diagnosis has witnessed a major shift for children in the last decades, with non‐biopsy approach becoming more and more frequent thanks to the last guidelines from the European Society of Pediatric Gastroenterology Hepatology and Nutrition (ESPGHAN).^11^ This resulted in a decreasing number of upper GI endoscopies performed in children for this clinical indication. Since 2020, indeed, a child/adolescent can be diagnosed just with two lab tests showing anti‐tissue transglutaminase IgA antibodies (TGA‐IgA) with values ≥ 10 times the upper limit of normal and positive anti‐endomysial antibodies (EMA‐IgA) in a second serum sample. Only patients not fitting these “antibody criteria” need to undergo biopsy confirmation during upper GI endoscopy under anesthesia. This important change led to avoid an invasive procedure in children and to receive at the same time an accurate diagnosis. Guidelines update also opened an unprecedented scenario for tackling climate change: if the diagnosis can be made just by these two laboratory tests, we can also reduce the hospital admissions, upper GI endoscopies, anesthesia, and related CO_2_ costs.

In this study, we focused on the environmental impact of this diagnostic shift for CeD in Umbria region, analyzing the current savings in CO_2_ emissions according to the different diagnostic pathways (without and with biopsy). In addition, we considered also CO_2_ emissions related to CeD follow‐up practices, to estimate the cumulative environmental burden of this disease.

## 2 METHODS

Patients aged between 0 and 18 years diagnosed and followed up for CeD in the Pediatric Clinic of Perugia University Hospital and in the Pediatric and Neonatology Unit of Foligno Hospital during 2020–2023 were included in the study. These two facilities are the only two referral centers for pediatric CeD at a regional level (Umbria region inhabitants, including both children and adults: 850,137 in January 2023). CeD patients were divided in two groups: Group 1 included patients diagnosed by upper GI endoscopy plus biopsies, while Group 2 included no‐biopsy patients.

Serological testing for TGA‐IgA performed at our centers was based on EliA test® (Termo Fisher Scientific), as well as in most public hospitals/community laboratories in our region; when this was not the case, those few assays which were not referenced for the “10xULN rule”^12^ were also centrally tested at our institution.

For each patient, the number of all the home‐to‐facility way and way back to achieve the diagnosis and the number of hospital outpatient consultations during the follow‐up have been considered.

These include: laboratory investigations requested by primary care, an outpatient visit to get scheduled for an endoscopy, blood tests before the upper GI, upper GI at the hospital, and an outpatient consultation to receive the final diagnosis and briefing on dietary requirements.

All parents from our study declared that they used their own or another relative's car to reach the hospital or the laboratory. No public transportation was used, given the peculiar geographic setting of the two hospitals and the Umbria region. The distance was estimated using the shortest geographical route from the patient's home address to the hospital sites or to the laboratory where the patients were tested for the diagnosis. The shortest geographical route from the patient's house, acquired from Google Maps (https://www.google.com/maps/), was considered, and it was assumed that everyone returned home directly after the blood sample or visit.

Given the current situation of electric and diesel vehicles in Italy, we have chosen to approximate the diesel vehicles. Transport emissions of CO_2_ per kilometer traveled by a diesel car were taken as reference and estimated as 171 g/km according to UK government source (https://ourworldindata.org/grapher/carbon-footprint-travel-mode). Upper GI endoscopy CO_2_ cost was estimated as 5.43 kg, and anesthesia‐related CO_2_ cost was estimated as 22 kg as per relevant literature.^13^

According to the ESPGHAN position paper on the management and follow‐up of children and adolescents with CeD,^14^ pediatric patients undergo a strict regimen of follow‐up: the first visit after the diagnosis is made in the following 6 months; subsequent visits are made every 6 months until normalization of TGA levels, and every 12–24 months thereafter.

Statistical analysis was performed using SPSS. The Shapiro–Wilk test was used to assess normality of data. Student T‐test was performed as appropriate, and a *p* < 0.05 was considered significant.

### 2.1 Ethics statement

The research protocol was approved by our IRB (Comitato Etico Territoriale dell'Umbria N° CE 1209/24). Informed consent was given by parents and/or patients.

## 3 RESULTS

Two‐hundred and thirty‐six patients diagnosed and followed up were included in the study. Group 1 included 43 patients diagnosed by upper GI plus biopsies, while Group 2 included 193 patients diagnosed according to nonbiopsy criteria.^11^

To achieve a biopsy diagnosis, the median number of routes in Group 1 was 10 and this resulted in a median CO_2_ cost/patient/year of 103 kg just as regards car emissions. In this group, the median CO_2_ cost/patient/year of the upper GI endoscopy and of anesthesia was also taken into account, which were 58.3 kg and 236.5 kg, respectively. The median cost/patient/year of cumulative CO_2_ product for the diagnosis of CeD by Upper GI endoscopy in the Umbria region was 397.9 kg.

The nonbiopsy diagnosis in Group 2 accounted for a median CO2 cost/patient/year of 57.2kg, and this value applies only to the number of home‐to‐facility way and way back from home to hospital/lab to achieve the diagnosis (Table 1). The difference of median CO_2_ emission/patient/year between the two groups was 340.7 kg (*p* < 0.001).

**Table 1:** Comparison of CO_2_ mean costs in children diagnosed with CeD by upper GI + biopsies (Group 1) and children diagnosed with CeD by nonbiopsy approach (Group 2).

	Group 1	Group 2	*p*‐Value
Number of patients	43	193	
Cars CO_2_ emissions (kg)	103	57.2	<0.001
Distance traveled in km	602.7	334.8	<0.001
Upper GI CO_2_ cost (kg)	58.3	/	
Anesthesia CO_2_ cost (kg)	236.5	/	
Total CO_2_ diagnosis cost (kg)	397.9	57.2	<0.001

Abbreviations: CeD, celiac disease; CO_2_, carbon dioxide; GI, gastrointestinal.

The follow‐up practices were the same for the two groups, with overlapping mean CO_2_ cost/patient/year (Group 1 40.5 kg vs. Group 2 38.1 kg; *p*: ns). This CO_2_ cost/patient/year was calculated according to the number of ways and ways back from home to lab and from home to hospital outpatient clinic where follow‐up consultations took place. These latter data are summarized in Table 2.

**Table 2:** Mean value of the distance traveled for the follow‐up/year/patient and the consequent CO_2_ mean cost.

	Group 1	Group 2	
Number of patients	43	193	
Distance in km	237	223.2	*p*: ns
CO_2_ cost (kg)	40.5	38.1	*p*: ns

Abbreviation: CO_2_, carbon dioxide.

## 4 DISCUSSION

We present here unprecedented data on CO_2_ emissions of pediatric CeD diagnostic and follow‐up pathways. Our study included patients from the Umbria region diagnosed and follow‐up in the last 4 years. As expected, when applicable, a non‐biopsy approach is extremely less impactful on the environment compared to biopsy diagnosis.

Our finding echoes the emerging perception that endoscopy conveys a relevant CO_2_ production^13^ and it is in line with the estimation about the CO_2_ burden of adult CeD diagnosed by means of endoscopy in the UK.^15^

According to our population data, the rate of diagnosis confirmed by biopsy is 22% (43/193). This finding may be attributable to the anti‐TGA‐IgA assay used in our setting, but it is consistent with other unpublished data. Patients from Group 1 who underwent endoscopy and biopsy confirmation had a median CO_2_ cost/patient/year of 397.9 kg, while Group 2 accounted only for 57.2 kg/patient/year. Most of the CO_2_ produced for Group 2 regards car emissions to go back and forth from the laboratory (twice) and from the outpatient setting where the diagnosis was made. Moreover, as the blood tests can be performed in any qualified/certified facility for this kind of assay, the family avoided a rather long way to the hospital (depending on the residence). Patients from Group 1 underwent a hospital admission and at least two outpatient consultations, whereas Group 2 traveled a much shorter distance since they have usually chosen a closer laboratory and finally came to the hospital outpatient clinic just to confirm the diagnosis, to receive the official report from the referral center confirming the diagnosis and to join the dedicated briefing on CeD and its dietary requirements on the same day. In our setting, patients with CeD undergoing upper GI endoscopy came to the hospital 3.5 times more than those receiving the “solo‐antibodies” diagnosis. These data are also partly attributable to the short watch and wait approach that is considered appropriate in patients with low TGA‐IgA and negative EmA. If possible on clinical grounds, in these cases children follow an unrestricted diet and repeat antibodies after few months: if still positive, upper GI endoscopy is then required.^16^ Moreover, the children of Group 1 had to come to the hospital not only for the blood tests before the upper GI (preadmission program), but also for the procedure itself and then, after histological examination, to receive a briefing on the diagnosis and its requirements.

On the contrary, the “solo‐antibodies” diagnosis not only speeds up the patient journey to treatment, but it also allows for the avoidance of an invasive procedure and strikingly cuts its environmental burden. Nevertheless, this study was not intended to suggest any easy shortcut to the non‐biopsy diagnosis for the sake of the environment. We are well aware of the heavy toll of CeD diagnosis and the mandatory need for diagnostic certainty. Should novel biomarkers enlarge the perspective for a 100% no‐biopsy approach, this will undoubtfully reduce the invasiveness of the procedure and positively impact on the climate change, for the sake of our children and the children of the future.

In our study, we also focused on CeD follow‐up practice, its environmental weight, and possible alternatives. After the Sars‐Cov‐2 pandemic, we witnessed a huge increase in telemedicine use. This new way of accessing healthcare services can have key impact on the environment. Carbon footprint savings related to telemedicine can range between 0.70 and 372 kg CO_2_ per consultation.^17^ If follow‐up were performed remotely, the same lab test data would be accessible, albeit a comprehensive physical examination would be impossible or at least still practicable to some extent with technical difficulties and regulatory barriers.^18^ This current analysis estimated the CO_2_ cost of the follow‐up of these patients and the hypothetical CO_2_ savings with a telemedicine follow‐up if applied in our setting. The follow‐up of the children diagnosed with CeD is a central issue of disease management, with the aims of ensuring a strict and well‐balanced gluten‐free diet, catch‐up growth in case of previous growth delay, and previous signs/symptoms remission. Follow‐up is also meant to identify other possible associated autoimmune disorders (for the most part, autoimmune thyroiditis and type‐1 diabetes mellitus) and, mostly in adults, disease complications. This follow‐up cannot avoid the laboratory investigations but—with some health education—it is imaginable a future with a central role of telemedicine in this setting, perhaps involving primary care physicians in close collaboration with pediatric gastroenterologists. Nowadays, in our real‐life experience, patients having their follow‐up at hospital outpatient clinic produce a median of 17 kg per year of CO_2_, but we are very far to propose telemedicine follow‐up without further prospective evidence on this issue. Suppose a telemedicine follow‐up is established in the very near future in our setting. In that case, it will be interesting to gather patient perspectives on this in advance, as it has been shown that patient‐centeredness might be sometimes perceived as weak.^19^ Theoretically, face‐to‐face interactions allow a better patient experience at least in the early stages of follow‐up, when a trustful patient‐physician relationship has to be built together, but this might not apply for longer periods.^20^ Perhaps a hybrid model, combining diverse patient needs and environmental sustainability, might be the most effective follow‐up pathway in the years to come.

We acknowledge a few limitations of our study. This is a regional study in which the findings can greatly depend on the intrinsic health organization and means of transportation of the region. Further studies in different centers are needed to evaluate the impact of the no biopsy approach on the CO_2_ emission in accordance with different hospital/regional frameworks. We did not consider newer and more sustainable car engines, still very limited in our setting. It is of note that laboratory tests and the biopsy processing/storage also have an environmental burden which were not considered as they were somewhat irrelevant in comparison to the other issues already evaluated in the study.^21^, ^22^, ^23^

## 5 CONCLUSION

The nonbiopsy approach for the diagnosis of CeD reduces invasiveness, health system costs, and has a conspicuous environmental benefit by decreasing CO_2_ emissions.

To our knowledge, this is the first pediatric work showing the positive impact on CO_2_ emission of no‐biopsy approach for CeD diagnosis. Whether the use of telemedicine for CeD follow‐up can have a promising role in the future remains to be established, albeit this also seems very effective from an environmental point of view.

## CONFLICT OF INTEREST STATEMENT

The authors declare no conflict of interest.
